# Interrelation Between Cerebrospinal Fluid Pressure, Intracranial Morphology and Venous Hemodynamics Studied by 4D Flow MRI

**DOI:** 10.1007/s00062-023-01381-0

**Published:** 2024-01-26

**Authors:** Florian F. Schuchardt, Axel J. Krafft, Lidia Miguel Telega, Sebastian Küchlin, Wolf A. Lagrèze, Theo Demerath, Philipp Arnold, Christian Fung, Luisa M. Kraus, Anja Hennemuth, Jürgen Beck, Horst Urbach, Cornelius Weiller, Andreas Harloff

**Affiliations:** 1 Department of Neurology and Neurophysiology, Medical Center—University of Freiburg, Faculty of Medicine, University of Freiburg, Freiburg, Germany; 2 Medical Physics, Department of Radiology, Medical Center—University of Freiburg, Faculty of Medicine, University of Freiburg, Freiburg, Germany; 3 Department of Neurosurgery, Medical Center—University of Freiburg, Faculty of Medicine, University of Freiburg, Freiburg, Germany; 4 Department of Neuro-ophthalmology, Medical Center—University of Freiburg, Faculty of Medicine, University of Freiburg, Freiburg, Germany; 5 Department of Neuroradiology, Medical Center—University of Freiburg, Faculty of Medicine, University of Freiburg , Freiburg, Germany; 6 Institute for Cardiovascular Computer-assisted Medicine, Charité, Universitätsmedizin Berlin, Campus Virchow-Klinikum, Berlin, Germany; 7 Department of Neurology and Neurophysiology, Medical Center—University of Freiburg, Faculty of Medicine, University of Freiburg, Breisacher Str. 64, 79106 Freiburg, Germany

**Keywords:** Idiopathic intracranial hypertension, Pseudotumor cerebri, Intracranial hypotension, Intracranial venous hemodynamics, Optic nerve sheath diameter

## Abstract

**Purpose:**

To quantify the effects of CSF pressure alterations on intracranial venous morphology and hemodynamics in idiopathic intracranial hypertension (IIH) and spontaneous intracranial hypotension (SIH) and assess reversibility when the underlying cause is resolved.

**Methods:**

We prospectively examined venous volume, intracranial venous blood flow and velocity, including optic nerve sheath diameter (ONSD) as a noninvasive surrogate of CSF pressure changes in 11 patients with IIH, 11 age-matched and sex-matched healthy controls and 9 SIH patients, before and after neurosurgical closure of spinal dural leaks. We applied multiparametric MRI including 4D flow MRI, time-of-flight (TOF) and T2-weighted half-Fourier acquisition single-shot turbo-spin echo (HASTE).

**Results:**

Sinus volume overlapped between groups at baseline but decreased after treatment of intracranial hypotension (*p* = 0.067) along with a significant increase of ONSD (*p* = 0.003). Blood flow in the middle and dorsal superior sagittal sinus was remarkably lower in patients with higher CSF pressure (i.e., IIH versus controls and SIH after CSF leak closure) but blood flow velocity was comparable cross-sectionally between groups and longitudinally in SIH.

**Conclusion:**

We were able to demonstrate the interaction of CSF pressure, venous volumetry, venous hemodynamics and ONSD using multiparametric brain MRI. Closure of CSF leaks in SIH patients resulted in symptoms suggestive of increased intracranial pressure and caused a subsequent decrease of intracranial venous volume and of blood flow within the superior sagittal sinus while ONSD increased. In contrast, blood flow parameters from 4D flow MRI did not discriminate IIH, SIH and controls as hemodynamics at baseline overlapped at most vessel cross-sections.

**Supplementary Information:**

The online version of this article (10.1007/s00062-023-01381-0) contains supplementary material, which is available to authorized users.

## Introduction

Cerebrospinal fluid (CSF) pressure changes directly affect pressure, volume and configuration of the other intracranial compartments, i.e., brain tissue, ventricles, intracranial veins and arteries (Monro-Kellie doctrine) [1]. Disabling complications related to intracranial pressure include vision loss due to optic neuropathy in idiopathic intracranial hypertension (IIH), and subdural hematoma in spontaneous intracranial hypotension (SIH). Both intracranial hypertension and hypotension are associated with distinct neuroimaging findings [2–4], including morphological changes of the intracranial venous system and the optic nerve sheath [5, 6]. Given the ease, accuracy and reproducibility of measurements by both transorbital sonography and ocular magnetic resonance imaging (MRI), the optic nerve sheath diameter (ONSD) serves as an indicator of intracranial pressure changes [5, 6]. Antidromic hydrodynamic effects let intracranial sinuses appear compressed and ONSD enlarged in IIH, and vice versa in intracranial hypotension [5–8]. IIH is associated with stenosis of the transverse-sigmoid junction, inducing local pressure gradients [7, 9–11]. Sinus stenting is thus increasingly promoted as an escalation step, using an invasive therapy for IIH patients with severe visual impairment, despite lacking evidence from randomized controlled trials [12].

Currently, our understanding of the interaction between CSF pressure and intracranial venous hemodynamics is limited. Two-dimensional time-of-flight (TOF) imaging offers a rapid overview of intracranial veins, derived from in-plane movement of spins during the acquisition phase. TOF imaging reflects endovascular flow hemodynamics and does not show venous morphology itself. For a long time, many intracranial veins were largely inaccessible by transcranial ultrasound or could only be studied by 2‑dimensional dynamic MR imaging [13–15]. The latter pointed toward reversible CSF pressure-associated hemodynamic effects in patients with IIH [13–15]. Hemodynamic studies in intracranial hypotension are currently lacking. Time-resolved three-dimensional phase-contrast MRI (4D flow MRI) enables noninvasive quantification and visualization of hemodynamics within any vessel within the body in vivo [16] without contrast application. Hence, the hemodynamic assessment of contiguous intracranial venous segments by 4D flow MRI may close this gap.

We aimed at analyzing the interaction of CSF pressure changes on intracranial venous volume and hemodynamics addressing the continuum of decreased to elevated intracranial pressure. We hypothesized that first, such changes can be demonstrated by multiparametric MR imaging, second, abnormal CSF pressure alters intracranial venous morphology and hemodynamics and, third, hemodynamic disparities are reversible when the underlying cause is resolved. We therefore compared cerebral venous volume, blood flow, and velocity in patients with IIH to age-matched and sex-matched healthy controls and patients with spontaneous intracranial hypotension. We chose IIH and SIH as both entities show extreme pressure levels, and we therefore expected to find distinct between-group differences even in a small cohort. In addition, we quantified morphological and hemodynamic changes in patients with spontaneous intracranial hypotension during the symptomatic phase and after clinical improvement due to neurosurgical closure of spinal dural leakages.

## Methods

### Ethics

The study received ethics board approval from University of Freiburg (Nr. 394/17) and conformed to the declaration of Helsinki. Prior to the start, we registered the study (German Clinical Trials Register No. DRKS00013741) and all participants gave written informed consent.

### Study Cohort and Design

We prospectively included patients between August 2018 and April 2021 from the Departments of Neurology, Neurosurgery and Neuro-ophthalmology of Medical Center, University of Freiburg. Recruitment was nonconsecutive due to restrictions during the COVID-19 pandemic.

### Eligibility Criteria

We screened therapy-naïve patients admitted for suspected IIH and SIH showing supportive paraclinical findings (elevated CSF opening pressure, papilledema on sonographic or ophthalmological examination in IIH; decreased CSF opening pressure or CSF leak on neuroimaging in SIH). We included patients older than 18 years with confirmed diagnosis of IIH or SIH following the diagnostic work-up described. Exclusion criteria were contraindications against MRI (e.g., claustrophobia, ferro-magnetic implants, pregnancy).

### Idiopathic Intracranial Hypertension

Patients with suspected IIH underwent study brain MRI based on clinical suspicion. IIH patients had to have new or worsening pre-existing headache, fulfilling the diagnostic criteria of IIH (ICHD‑3, 7.1.1) [17] and CSF pressure exceeding 25 cmH_2_O with pulsatile tinnitus and/or papilledema, not better accounted for by another ICHD‑3 diagnosis [17]. We included patients with recently diagnosed IIH prior to treatment and 9/11 IIH patients underwent study brain MRI before lumbar puncture (LP).

### Controls

We recruited volunteers matched by age and sex to previously included IIH patients. Healthy controls had to lack headache of unknown etiology, prior history of intracranial hypotension or hypertension, CSF shunt, or optic neuritis.

### Spontaneous Intracranial Hypotension

Patients with SIH underwent the study MR protocol after the diagnosis was established according to ICHD‑3 criteria [17]. Patients with SIH had to have headache developing in temporal relation to low CSF pressure or CSF leakage, present low CSF pressure (< 6 cm H_2_O) and/or evidence of CSF leakage on imaging, with signs and symptoms not better accounted for by another ICHD‑3 diagnosis [17]. In line with ICHD‑3 criteria, we omitted intracranial pressure measurement by LP confirming CSF opening pressure < 6 cm H_2_O when the clinical presentation was typical and a CSF leak was objectified on spinal imaging in MRI and/or myelography (ICHD‑3, 7.2.3) [17]. All SIH patients either had a spinal bone spur (type 1 CSF leak due to dural tear) or a CSF cyst (type 2 CSF leak due to meningeal diverticula) according to the classification of Schievink et al. [18] with consecutive dural leakage in spinal imaging and underwent neurosurgery for leak closure [19].

### Cerebrospinal Fluid Opening Pressure Measurement

Supplementary Table 1 shows CSF opening pressure in individual IIH and SIH patients. Controls did not undergo LP as it was deemed unethical without indication.

### Neuroimaging

Brain MRI was performed using a 3T scanner (PRISMA, Siemens Healthineers, Erlangen, Germany) with a 32 channel head coil. All participants underwent the identical protocol at baseline, including intracranial venous hemodynamic assessment by 4D flow MRI, TOF and a T2-weighted half-Fourier acquisition single-shot turbo-spin echo (HASTE) sequence imaging optimized for quantification (Table 1). To examine dynamic changes in patients with SIH following surgery, this group underwent follow-up MRI after clinical response to treatment (Table 2).

**Table 1:** MR imaging protocol

Acquisition	Parameter
TOF	FOV = 250 × 250 × 110 mm^3^, TR/TE = 21/4.83 msec, voxel size = 0.8 × 0.8 × 2.5 mm^3^
4D Flow MRI	FOV = 210 × 210 × 72 mm^3^, TR/TE = 94.8 ms/5.0 ms, flip angle = 15°, velocity sensitivity = 0.4 m/s, spatial/temporal resolution = 0.9 × 0.9 × 0.9 mm^3^/94.8 ms, acceleration factor = 5, mean cycle = 655 ± 125 ms, mean acquisition time = 12:38 min
HASTE	FOV = 210 × 184 mm^2^, TR/TE = 1680/127 ms, bandwidth = 196 Hz/pixel, echo train length = 9 ms, spatial resolution = 0.4 mm^2^, slice thickness = 2 mm

*TOF* time-of-flight, *HASTE* T2-weighted half-Fourier acquisition single-shot turbo-spin echo

**Table 2:** Aggregated participants’ demographics

	IIH (*n* = 11)	Control (*n* = 11)	SIH (*n* = 9)
	Mean ± SD		
Age (years)	31.5 ± 9.9	31.7 ± 10.7	43.2 ± 7.6
Sex	9 female, 2 male	9 female, 2 male	4 female, 5 male
Height (m)	1.72 ± 0.06	1.71 ± 0.08	1.79 ± 0.07
BMI (kg/m^2^)	36.1 ± 6.7	26.9 ± 8	25.3 ± 5.2
CSF opening pressure (cmH_2_O)	40.7 ± 8.9	n. a.	11.9 ± 8.1 (*n* = 8)
Time between baseline MRI and LP (days)	2.5 ± 4.3, MRI before LP	n. a.	1.9 ± 3.2, LP before MRI
Latency baseline to F/U MRI (days)	n. a.	n. a.	211.9 ± 288
Latency operation to F/U MRI (days)	n. a.	n. a.	167.1 ± 237.6

Complete data were available, if not indicated otherwise

*BMI* body mass index, *CSF* cerebrospinal fluid, *IIH* idiopathic intracranial hypotension, *n.* *a.* not applicable, *SD* standard deviation

### Neuroimaging Analysis

To avoid bias and ensure blinding of readers, we separated the collection and analysis of both imaging and demographic data, which we pseudonomized and kept in separate tables. We anonymized identifiable MR data prior to analysis by replacing names and family names by generic IDs and acquisition dates by random pseudo-dates using the programs’ built-in anonymization tool (IMPAX EE R20 DeepUnity Diagnost, Dedalus HealthCare GmbH, Bonn, Germany). This made identifying participants by the search within the patient data management system impossible. To avoid memorization, we analyzed MR data with a few weeks distance. We measured anonymized ONSD in the sequential order of the pseudo-date, which hence represented a randomized sequence. Finally, we re-attributed anonymized results to pseudonymized data (group affiliation) only after completing analyses of all datasets of all groups.

### Volumetric Assessment of Intracranial Veins

We quantified volumetric differences between groups and within SIH patients before versus after surgery using an in-house built post-processing platform (NORA, www.nora-imaging.org). Volumetry of large intracranial venous vessels was based on minimal intensity projection of 2D TOF venographies (Table 1). The segmentation relied on the maximum intensity differences of voxels between the time-of-flight angiogram and the surrounding tissue. The superior sagittal sinus (SSS), straight and both transverse sinuses were semi-autonomously segmented in 3 steps: first, we performed skull stripping of the 3D-TOF-images using the Brain Extraction Tool [20]. We set the fractional intensity threshold (F) to 0.3 and the vertical gradient in fractional intensity threshold (G) to −0.3. Second, we retrieved veins and sinuses from the skull-stripped 3D-TOF-images by using a threshold-based segmentation (threshold 0.4). We normalized the maximum intensity to 1 and labelled voxels above 12.5% of the maximum signal intensity as belonging to the veins/sinuses. Last, a physician with 2 years experience in neuroimaging visually validated or corrected results where necessary.

To consider both the influence of head size on the dimensions of the intracranial veins and sinuses and to make interindividual comparisons more reliable, we normalized the absolute TOF volumetry (mm^3^) of intracranial sinuses using the individual brain volume (mm^3^) as the divisor. Using SPM 12 (https://www.fil.ion.ucl.ac.uk/spm/software/), we segmented 3D TOF datasets into compartments. We then calculated a brain mask based on the sum of C1 (grey matter) and C2 (white matter), yielding the sum of all voxels within the volume.

### Hemodynamic Assessment of Intracranial Veins

**Fig. 1:**
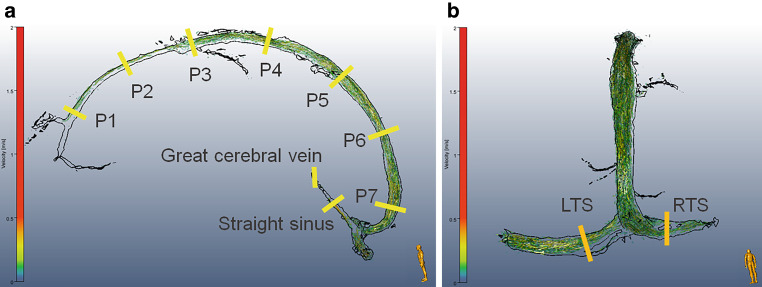
Visualization of intracranial venous flow using 4D Flow MRI in birds’ view perspective. The systematic analysis includes seven equidistant cross-sections within the superior sagittal sinus (P1–P7), and one cross-section within the great cerebral vein (vena cerebri magna, VCM), straight sinus (**a** sagittal view), left (LTS) and right (RTS) proximal transverse sinus (**b** coronal view). Color coding represents the velocity [m/s] of flow trajectories

We visualized and quantified intracranial venous hemodynamics (i.e., mean blood flow and velocity) using MEVISflow (version 10.2, Fraunhofer MEVIS, Bremen, Germany) [21, 22]. One reader, a master thesis student with over 12 months experience in 4D flow analysis of aortic and carotid MR datasets, analyzed anonymized datasets in random order to avoid bias. Preprocessing steps included automated Eddie current correction [23] and phase-unwrapping [24]. In the watershed-based segmentation, we used a mask threshold height of 1–2 for the SSS and transverse sinuses and 0.4–0.8 for straight sinus and great cerebral vein (vena cerebri magna, VCM). We systematically assessed blood flow information based on 4D flow MRI angiography at the 11 distinct venous vessel segments depicted in Fig. 1 as described previously [25]. The systematic prespecified analysis of venous hemodynamics was not feasible in single cases, within SSS at P1 (late conjunction of frontal bridging veins: IIH6, HC10), and P7 (early bifurcation of SSS in RTS and LTS; IIH1), at VCM (insufficient flow-tissue contrast: HC10, SIH1, 3 at baseline), RTS (aplasia: HC3) and LTS (aplasia: IIH4, 6, 9; HC1, 5; SIH3, 6).

### MRI of the Optic Nerve Sheath

We measured optic nerve sheath diameter (ONSD) of both eyes using a HASTE sequence (Table 1) as previously described [26]. In short, we measured ONSD 3 mm behind the optic nerve head and oriented perpendicular to the longitudinal extension of the optic nerve using a commercial ROI tool (IMPAX EE R20 DeepUnity Diagnost, Dedalus HealthCare GmbH). We defined the border of the optic nerve sheath as the zone of maximum contrast difference between the hyperintense T2 signal of CSF and the surrounding soft tissue.

### Statistics

Continuous data are presented as mean ± standard deviation (SD) and partitions in percentage. For analyses of categorical variables, we used χ^2^ and Fishers’ exact tests as appropriate. We analyzed continuous variables using simple t‑test for cross-sectional between-group analyses and paired t‑test for longitudinal within-group analyses. To account for the inclusion of both eyes, we calculated the population mean estimates ± standard error of the ONSD using mixed effects linear models with random effects per eye nested within patients. We assessed correlations of continuous variables by linear regression analysis. All tests were two-sided (α-level 5%). Because of the exploratory nature of the study, descriptive *p*-values and 95% confidence intervals are neither corrected for multiple comparisons nor controlled for confounding. We assumed that missing data occurred at random and did not impute. We prepared figures using SPSS version 27 ((IBM, Armonk, NY, USA) and R (The R Project for Statistical Computing, version 3.6.2; nlme package, version 3.1-153; https://www.r-project.org/).

## Results

### Cohort

Table 2 summarizes aggregated patient information and time intervals between LP, MRI and surgery. Supplementary Table 1 shows detailed individual patient data. Neuroimaging of controls was normal. The CSF opening pressure differed significantly between IIH and SIH patients (*p* < 0.001) but was not available in one SIH patient (SIH 6, “dry tap” at lumbar puncture). Postoperatively, following the closure of CSF leaks, 3 patients (SIH 5, 6, 8) experienced typical symptoms of intracranial hypertension, 5 patients with intracranial hypotension underwent early postoperative MRI within 3.4 ± 1.5 days (95% confidence interval, CI 2–4.5) after closure of the CSF leak, the remaining 4 underwent MRI 371.8 ± 223.7 days (95% CI 170.3–540) after surgery.

### Volumetry of Cerebral Sinus

**Fig. 2:**
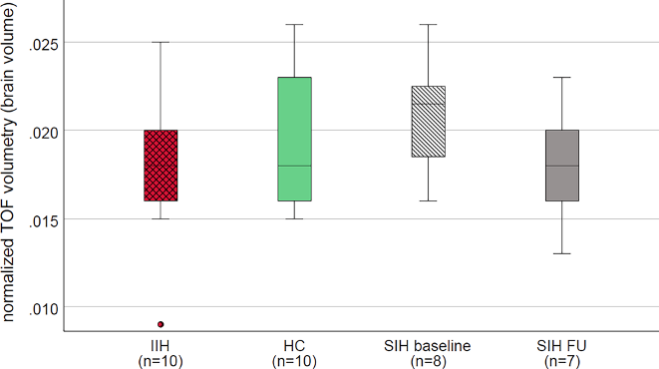
Boxplots show normalized time-of-flight (TOF) volumetry of the large intracranial sinus and deep intracranial veins. Between-group differences in idiopathic intracranial hypertension (IIH), age-matched and sex-matched healthy controls (HC) and patients with spontaneous intracranial hypotension (SIH) at baseline were not significant (simple t‑test; IIH vs. HC, *p* = 0.454; HC vs. SIH baseline, *p* = 0.360; IIH vs. SIH baseline, *p* = 0.125), yet patients with SIH showed a trend towards a reduction of intravenous volume after treatment (dependent t‑test, *p* = 0.067). *FU* follow-up

Figure 2 presents volumetry of intracranial veins and large sinuses by group, and longitudinal changes in SIH patients, normalized by the individual brain volume. At baseline, t‑test yielded comparable volumes between groups (HC versus IIH, *p* = 0.454; HC versus SIH, *p* = 0.360), showing a trend towards higher volume in SIH versus IIH (*p* = 0.125, 95% CI −0.007 to 0.001). In addition, after treatment of intracranial hypotension, SIH patients showed a trend towards lower intrasinus volume (*p* = 0.067, 95% CI −0.0002 to 0.005). Venous volume was lowest in SIH patients after treatment of intracranial hypotension, going along with symptoms of postinterventional intracranial hypertension in several patients. We excluded four datasets (incomplete coverage of the sinus tree, SIH1 at baseline and IIH5; normalization not feasible due to hygroma, SIH4 at follow-up; noisy MRI, HC10).

### Intracranial Venous Hemodynamics

**Fig. 3:**
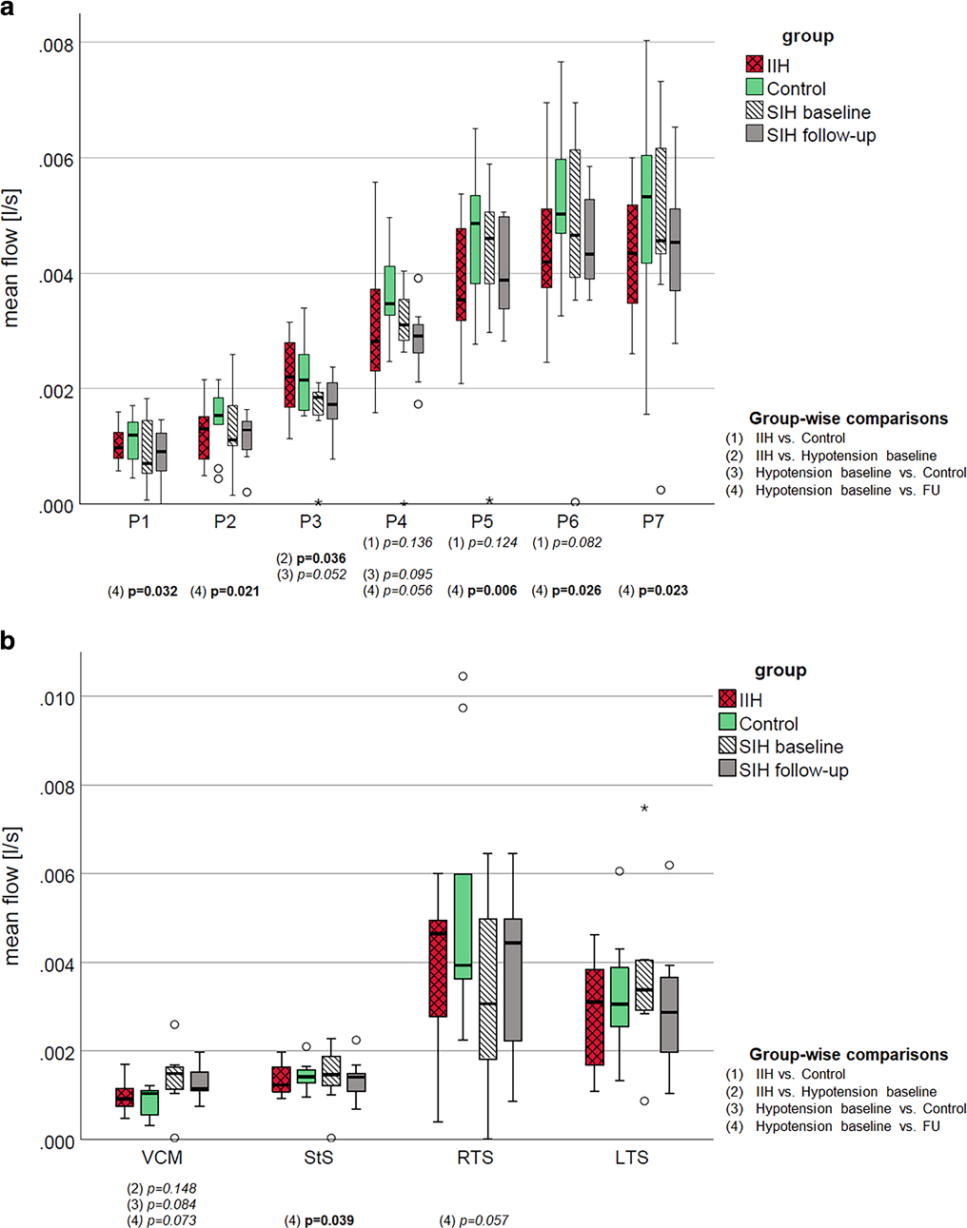
Boxplots demonstrate venous blood flow at cross-sections within the superior sagittal sinus (**a**; P1 is located rostral, P7 dorsal), and in the great cerebral vein (VCM), straight (StS), right (RTS) and left (LTS) transverse sinuses (**b**). *P*-values below the cross-sections give between-group differences at baseline (t-test) and longitudinal within-group changes at baseline versus after treatment in patients with intracranial hypotension (dependent t‑test). Statistically significant differences (*bold*) and trends (*italics*) are indicated below the graph. *FU* follow-up, *IIH* idiopathic intracranial hypertension, *LTS* left transverse sinus, *RTS* right transverse sinus, *StS* straight sinus, *VCM* vena cerebri magna

Figure 3 depicts mean blood flow at the intracranial venous cross-sections, stratified cross-sectionally by group, decreased (SIH), normal (control) and elevated intracranial pressure (IIH) at baseline and SIH after surgery. Between groups, mean blood flow differed visually without reaching significance at the investigated segments at baseline, except for significantly lower flow at P3 in SIH compared with IIH. Groups with higher intracranial pressure (IIH versus controls; SIH following treatment versus before) showed overall visually lower blood flow, reaching statistical significance only in the longitudinal comparisons of SIH patients. In all SSS segments, flow was highest in controls, except for comparable flow at P3 in IIH. Following treatment of SIH, mean flow within the middle and dorsal SSS and within deep intracranial veins decreased compared with baseline, except for increased flow within the frontal SSS (P1–P2) and the right transverse sinus at follow-up and comparable mean flow in the left transverse sinus.

Supplementary Figure 1 shows the distribution of mean velocity at the respective segments. Overall, velocity did not differ between groups at baseline (except for a trend towards lower velocity in IIH versus controls at the dorsal SSS, P5 and P6, and higher velocity at VCM in IIH and SIH versus controls) or longitudinally in SIH patients (before versus after neurosurgery).

### Optic Nerve Sheath Diameter

**Fig. 4:**
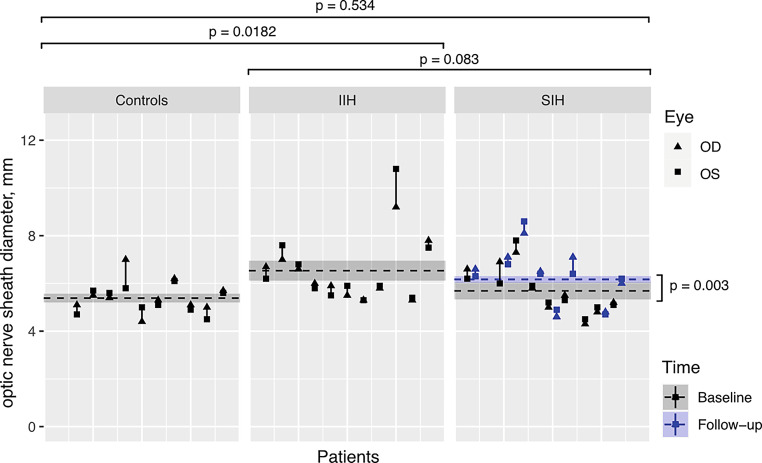
Optic nerve sheath diameter by group, used as a surrogate of CSF pressure in controls (*left*), patients with idiopathic intracranial hypertension (IIH, *middle*), and spontaneous intracranial hypotension (SIH, *right*), including longitudinal dynamic changes in SIH patients before (baseline, *black*) versus after treatment (follow-up, *blue*). Vertical lines depict within-patient measurements of the right eye (triangles; OD, oculus dexter) and left eye (*squares*; OS, oculus sinister). We present population mean estimates as dashed horizontal lines. The shaded areas are population mean estimates ± the standard error of the mean. *P*-values are based on mixed effects linear models with random effects per eye, nested within patients

Optic nerve sheath diameter (Fig. 4) was overall larger in patients presenting symptoms of higher CSF pressure (IIH, SIH post-treatment) compared with controls and SIH patients before treatment (reaching significance in IIH versus controls (6.5 ± 0.4 mm versus 5.4 ± 0.2 mm (mean ± standard error), *p* = 0.018) and SIH postoperative versus preoperative (6.2 ± 0.1 mm versus 5.7 ± 0.4 mm, *p* = 0.003)). By contrast, we did not observe significant differences in ONSD between patients with IIH and SIH in this cohort. Following treatment, ONSD increased significantly in patients with SIH. ONSD was not quantifiable in HC1 at baseline and in SIH7 at follow-up due to bilateral insufficient ONSD-soft-tissue contrast.

### Association Between Venous Volumetry and Cerebrospinal Fluid Opening Pressure

The CSF opening pressure and TOF volumetry did not correlate significantly (*p* = 0.79).

## Discussion

Our main findings are: first, interrelations between intracranial pressure alterations and morphological changes of ONSD were demonstrable both cross-sectionally and longitudinally using multiparametric brain MR. Second, SIH treatment by neurosurgical leak closure resulted in lower intravenous volume, significantly higher ONSD going along with the resolution of SIH symptoms or development of overshooting symptoms of intracranial hypertension suggesting increase and/or normalization of CSF pressure. This effect showed a trend towards discriminating groups, with remarkable longitudinal differences in the intracranial hypotension group following treatment of intracranial hypotension. Third, within the SSS venous blood flow at baseline differed significantly only at the middle segment P3 between groups. Following treatment of intracranial hypotension, patients showed lower mean blood flow within the middle and dorsal superior sagittal sinus (SSS) and the deep intracranial veins, stable flow at the left transverse sinus, yet increased flow at the right transverse sinus. Mean velocity was overall comparable between groups both at baseline and follow-up. Finally, the ONSD, used as a surrogate for intracranial pressure, was larger in IIH patients than in both matched controls (significant) and patients with intracranial hypotension before interventional treatment (trend). The ONSD increased significantly in patients with intracranial hypotension following closure of CSF leaks, accompanying symptoms of intracranial hypertension in several patients.

Taken together, CSF pressure changes showed longitudinal effects on intracranial morphology, venous volume and hemodynamics. Treatment of intracranial hypotension decreased intracranial venous volume, reduced flow within the dorsal two thirds of the SSS and increased ONSD.

In IIH, a close, positive correlation between CSF pressure and body mass index is well established [27] and MR data suggest impaired CSF homeostasis with increased extraventricular CSF volume [13]. CSF pressure-related disease interrelate with hemodynamics. Also, cerebrospinal pressure alterations bear on the intracranial venous system morphology, as comparison of venous volumetry in SIH patients before versus after surgery confirmed. The elevated intracranial pressure in IIH compresses intracranial sinuses [7] and promotes the formation of stenoses, most frequently (93%) at the transverse-sigmoid junction [9–11]. Stenoses regressed or normalized following CSF pressure reduction by lumbar puncture in several previous studies [11, 28–30], although others reported static narrowing following LP [31].

In contrast to IIH, sinuses appear distended in intracranial hypotension [7, 8]. The enlargement of intracranial veins observed in SIH patients at baseline might induce venous blood pooling and secondary flow reduction within the SSS (see below). In line, low CSF pressure treatment by CSF leak closure led to decreasing intracranial vein and sinus volumes in SIH patients, comparable to the compression of sinuses in IIH. Following treatment, intravenous volume in SIH decreased to a level comparable to IIH patients. We believe that this reflects the overshooting effect of CSF leak closure on intracranial pressure early after the intervention because it fitted with occurrence of symptoms of intracranial hypertension in a number of patients. It is plausible that the previously compensatory increase of CSF production needs time to adapt to the rising intracranial pressure. Notably, we observed venous volume changes, although half of the patients underwent follow-up several months after the intervention. Because of the small sample size, we omitted comparisons between patients undergoing early versus late follow-up MRI. Intracranial sinus volumes showed a trend towards enlargement in SIH, but based on time-of-flight venography, did not differ between groups at baseline and therefore is not suitable as an isolated discriminator in individual patients. Time-of-flight angiography, relying on inflow phenomena, might have insufficient sensitivity to detect small volume differences. Nevertheless, the trend towards lower volumes in IIH versus SIH and longitudinal intraindividual comparisons within the same SIH patients before and after surgery at baseline plausibly demonstrates the effects of CSF pressure differences as expected from the Monro-Kellie doctrine. In line, despite the small sample size, flow within the SSS and straight sinus was lower in SIH.

Using 4D flow MRI, we were able to quantify hemodynamic effects of cerebrospinal pressure alterations. Both the compression of the sinus tree and localized sinus stenoses impede venous outflow. Focal stenoses in IIH create local endovenous pressure gradients [9, 32–34]. As 4D flow MRI indicates, the transstenotic velocity at transverse sinus stenoses correlates with venous pressure gradients [35]. Starting around 30% lumen narrowing, endoluminal pressure rises 3.5 mm Hg for every 10% stenosis [32].

Our findings add to previous observations of focal hemodynamic differences using isolated 2D phase-contrast imaging planes in IIH [14]. Given the ubiquitous hydrostatic effect of CSF pressure elevation on intradural sinuses, our findings are in line with the hypothesis of a generalized interaction between intracranial CSF hydrostatics and venous hemodynamics. Following the closure of CSF leaks in the SIH group, we observed the same effects reflecting CSF pressure elevation as found in IIH patients (i.e., compression of intracranial veins [7] quantified by regredient intracranial venous volume, flow reduction [36] and increasing ONSD [5]). Overall, between-group differences at baseline did not reach significance in our cohort, possibly due to the small sample size. The lack of discrimination of venous volumes between groups might result from hemodynamic effects of time-of-flight imaging rather than reflecting anatomic volumetric difference. Future studies using alternative techniques, e.g., high-resolution phase-contrast or contrast-enhanced morphological imaging, might address this aspect.

The currently published data supporting flow alterations in CSF pressure-related disease is inconclusive. Yet, several clues point toward effects of intracranial pressure changes on intracranial venous hemodynamics. Patients with symptomatic intracranial hypotension showed an increased diameter and maximum flow velocity within the superior ophthalmic vein; the veins’ flow direction reversed after intrathecal pressure normalization following epidural blood patch and resolution of symptoms [37]. IIH patients presented antidromic findings: compared with controls, cerebral blood transit time on contrast-enhanced duplex ultrasound was prolonged [36] and 2D flow-sensitive phase-contrast MRI showed reduced flow in the superior sagittal sinus [14] and extracranial veins [13]. In IIH patients with tinnitus, intracranial pressure reduction by lumbar puncture led to regredient flow velocity over stenoses on 4D flow MRI [38], supporting their reversibility. Yet, the adaptation of hemodynamics to intracranial pressure changes might be time-dependent and they may occur before the amelioration of symptoms [28].

Partially contrasting these observations in adults, Bateman et al. found a 46% increase of total blood flow within the SSS of pediatric IIH patients (where IIH due to hyperemia is more frequent than in adults [15], who show stenoses in > 90% [39]) and 25% reduced blood flow within the SSS in patients presenting transverse sinus stenoses [40]. Further groups found comparable hemodynamics in adult IIH patients versus controls [41, 42].

Both elevated and decreased CSF pressure induce changes of ONSD [5, 6]. In SIH, ONSD increased and symptoms of intracranial hypotension abated postinterventionally in most patients, even causing symptoms of intracranial hypertension in some. Complementing ONSD enlargement, postinterventional flow within the SSS decreased to levels similar to IIH patients. Contrasting previous data [6], the ONSD in hypotensive patients before treatment did not differ from non-matched controls, possibly due to an outlier (patient SIH 3) in this small cohort.

Given the limited sample size of this exploratory pilot study, our results must be considered with caution and need reproduction in larger cohorts. As we recruited patients presenting to neurology, neurosurgery and neuro-ophthalmology services, we might have selected IIH patients with a predominance of headache and visual symptoms, possibly underrepresenting patients with pulsatile tinnitus or other symptoms. We did not obtain follow-up MRI data of IIH patients, as almost all had elevated CSF opening pressure in lumbar puncture at repeated control visits, despite medical treatment. The timing of cerebral MR follow-up in SIH patients possibly introduced heterogeneity. In addition, the inclusion of a majority of SIH patients with intracranial hypotension due to bone spurs limits the generalizability of our results to other causes of intracranial hypotension such as leaking nerve root casts, and CSF venous fistulas. The limited field of view of the phase-contrast sequence only captured the proximal transverse sinuses. Unfortunately, in-plane artifacts appearing in the non-contrast-enhanced TOF venogram hindered determining the prevalence of transverse sigmoid sinus stenoses in participants.

## Conclusion

CSF pressure and intracranial venous hemodynamics are interrelated. Using MRI including 4D flow MRI in homogeneous cohorts of IIH patients before lumbar puncture, age-matched controls and SIH patients with dural leaks, we were able to demonstrate and quantify the consequences of CSF pressure changes on intracranial venous volume, hemodynamics, and ONSD. Intracranial venous volume showed an inverse relation with intracranial pressure while hemodynamics did not discriminate groups at baseline, given the overlap of flow at most sinus segments. Longitudinal flow within the dorsal two thirds of the superior sagittal sinus decreased following treatment of CSF leaks, whereas velocity remained overall unchanged. In contrast, higher intracranial pressure, both in IIH and in SIH following CSF leak closure, was associated with ONSD increase. Taken together, 4D flow MRI adds to our pathophysiologic understanding, but currently does not complement the diagnostic spectrum in CSF pressure-related disease, in contrast to ONSD.

## What is Known on this Subject?

- Idiopathic intracranial hypertension (IIH) may be caused by elevated intrasinus resistance due to outflow restriction due to stenoses (> 90% in adults), elevated extracranial venous pressure, cerebral hyperemia, or increased CSF production.
- Elevated intracranial pressure translates to dilated optic nerve sheaths.
- Previous analyses performed at isolated vessel segments in IIH point towards reversible hemodynamic alterations that respond to CSF pressure reduction.

## What Does this Paper Add?

- Systematic hemodynamic assessment of superficial and deep intracranial veins using 4D flow MRI in decreased, normal and elevated intracranial pressure.
- Effects of CSF pressure changes on venous volume and hemodynamics are reversible, showing opposed directions in SIH and IIH patients.
- Evidence of an extended, incremental frontodorsal flow reduction within the superior sagittal sinus in patients with intracranial hypotension following closure of CSF leaks, partly showing symptoms of postoperative intracranial hypertension.

### Supplementary Information

Supplementary Table 1 Patients’ demographics. *Asterisk* patients’ description. *C* cervical,* CSF* cerebrospinal fluid, *IIH* idiopathic intracranial hypotension, *N. VI.* sixth cranial nerve, *SIH* spontaneous intracranial hypotension, *th* thoracic. In SIH patients, the type of spinal CSF leak is classified according to Schievink et al. [18].

Supplementary Fig. Boxplots depict venous flow velocity at cross-sections within the superior sagittal sinus (a; P1 is located rostral, P7 dorsal), and in the great cerebral vein (VCM), straight (StS), right (RTS) and left (LTS) transverse sinuses (b). Between-group comparisons did not show significant different velocities (simple t‑test). Longitudinal within-group changes in intracranial hypotension patients at baseline versus after treatment did not reach significance (dependent t‑test). Trends (italics) are indicated below the graph. *FU* follow-up, *IIH* idiopathic intracranial hypertension
