# Supplementary material for: Interrelation Between Cerebrospinal Fluid Pressure, Intracranial Morphology and Venous Hemodynamics Studied by 4D Flow MRI
Source: Clin Neuroradiol. 2024 Jan 26;34(2):391–401. doi: 10.1007/s00062-023-01381-0 (PMC11130051; doi:10.1007/s00062-023-01381-0)
Supplement: Supplementary file 1 — Supplementary Table 1 Patients’ demographics. Asterisk patients’ description. C cervical, CSF cerebrospinal fluid, IIH idiopathic intracranial hypotension, N. VI. sixth cranial nerve, SIH spontaneous intracranial hypotension, th thoracic. In SIH patients, the type of spinal CSF leak is classified according to Schievink et al. [18]. [file 62_2023_1381_MOESM1_ESM.docx]

| **ID** | **Sex** | **Age** [years] | **Onset of symptoms; clinical features** | **Duration** | **Neuroimaging** | **CSF opening pressure**  [cm H_2_O] | **Treatment** |
| --- | --- | --- | --- | --- | --- | --- | --- |
| IIH1 | female | 47 | Progressive, left sided headache, blurring left eye; supine worse, best upright; bilateral papilledema | 7 months | Bilateral stenosis of transverse-sigmoid sinus, pressure gradients 13-22mmHg over stenoses | 30 | Acetazolamide, Topiramate, Furosemide; repetitive lumbar puncture |
| IIH2 | female | 23 | Subacute progressive headache, left N. VI paresis; bilateral papilledema | 1 week | Bilateral focal transverse sinus stenosis, partially empty sella | 50 | Acetazolamide; repetitive lumbar puncture |
| IIH3 | female | 25 | Subacute progressive headache, diplopia, right N. VI paresis; bilateral papilledema | 6 days | Bilateral small sized transverse sinus (right side hypoplastic), bilateral optic disc protrusion, partially empty sella | 50 | Acetazolamide; repetitive lumbar puncture |
| IIH4 | female | 24 | Blurred vision, medium intense left frontal headache; bilateral papilledema | 6 weeks | Hypoplastic left transverse sinus, optic disc protrusion, partially empty sella | 34 | Acetazolamide; lumbar puncture |
| IIH5 | female | 33 | Daily, numb, fluctuating headaches; intermittent visual deficits after stooping down; bilateral papilledema | 8 weeks, visual troubles 4 weeks | Bilateral transverse sinus stenosis, bilateral optic disc protrusion, partially empty sella | 47 | Acetazolamide; repetitive lumbar puncture |
| IIH6 | female | 35 | Visual blurring, migraine-type headache without positional change; bilateral papilledema | 9 months | Focal stenosis of right transverse sinus (Pacchioni granulation), hypoplastic left transverse sinus | 30 | Acetazolamide; lumbar puncture |
| IIH7 | female | 28 | Daily persistent headache, worsening in supine position; bilateral papilledema | 6 months | Bilateral transverse sinus stenosis, partially empty sella | 38 | Acetazolamide; lumbar puncture |
| IIH8 | male | 21 | Blurred vision, progressive headache, nausea, phonophobia; bilateral papilledema | 3 weeks | High-grade stenosis of left transverse-sigmoid sinus, hypoplasia of right transverse sinus; | 28 | Acetazolamide, Topiramate; repetitive lumbar punctures; implantation of Rickham reservoir; left transverse sinus stenting after failure of conservative measures |
| IIH9 | female | 29 | Chronic headaches; increasing frequency and positional dependence during last year with intermittently blurred vision; bilateral papilledema | 12 months | Left hypoplastic transverse sinus, partially empty sella | 47 | Acetazolamide, Metformine |
| IIH10 | female | 52 | Decreased vision, photopsia; no headache; bilateral papilledema | 6 weeks | Bilateral transverse sinus stenosis due to Pacchioni granulations, partially empty sella | 44 | Acetazolamide; lumbar puncture |
| IIH11 | male | 28 | Unilateral left temporal visual field deficit, left-sided headache; bilateral papilledema | 1 week | Bilateral distal transverse sinus stenosis, partially empty sella | 50 | Acetazolamide; repetitive lumbar puncture |
| SIH1 | female | 41 | Acute, posture dependent headache, intractable when upright | 6 months | Ventral CSF leak (type 1), bone spur, vertebrae th 1/2 | 15 | Several blood patches, operative closure of 12 mm dural cleft |
| SIH2 | male | 55 | Acute, severe orthostatic headache | 6 weeks | Ventral CSF leak (type 1), bone spur, vertebrae th 3/4 | 15 | Blood patches, operative closure of 5 mm dural cleft |
| SIH3 | male | 37 | Chronic, orthostatic hedache | 3 years | Ventral CSF leak (type1), bone spur, vertebrae th 8/9 | 13 | Current: resection of bone spur, covering of 6mm ventral dural cleft; previous: blood patches, covering of fistula C6-root (right), gathering of dural cyst lumbar vertebrae 2/3 |
| SIH4 | male | 46 | Acute, orthostatic nuchal and headache | 5 months | Ventral CSF leak (type1), bone spur, vertebrae th 1/2; left fronto-temporo-parietal subdural hematoma | 29 | Operative closure of 5mm ventral dural cleft |
| SIH5 | female | 46 | Acute, orthostatic headache, nausea and nuchal rigidity;  postoperative symptoms of intracranial hypertension | 6 months | dorsal meningeal diverticulum, type 2 CSF leak, vertebrae th 5/6 | 8 | Operative closure of 2mm dural cleft of a dehiscent dural cyst; postural headache increasing when supine during the first days after operation |
| SIH6 | male | 39 | Acute, thunderclap headache, right N. VI paresis; postoperative symptoms of intracranial hypertension | 6 weeks | Ventral CSF leak (type1), bone spur, vertebrae th 1/2 | punctio sicca | High-volume blood patch, operative closure of 11mm ventral dural cleft; postural headache increasing when supine during the first days after operation |
| SIH7 | female | 34 | Acute, intractable orthostatic headache and tinnitus, “brain fog”* | 8 weeks | Ventral CSF leak (type1), bone spur, vertebrae th 12/ lumbal 1 | 10 | Operative closure of 2mm ventral dural cleft |
| SIH8 | male | 54 | Acute orthostatic headache, nausea, emesis, hearing troubles; postoperative symptoms of intracranial hypertension | 4 months | Ventral CSF leak (type 1), bone spur and calcified disc protrusion, vertebrae th 7/8; bilateral fronto-temporo-parietal subdural hematoma | 4 | Operative closure of ventral dural cleft, resection of disc calcification; postural headache increasing when supine during the first days after operation |
| SIH9 | female | 37 | Acute orthostatic headache | 9 months | Ventral CSF leak (type 1), bone spur, vertebrae th 12/ lumbal 1 | 10 | Operative closure of 5mm ventral dural cleft |

**Supplementary Table 1** Patients’ demographics. *patients' description. C, cervical; CSF, cerebrospinal fluid; IIH, idiopathic intracranial hypotension; N. VI., sixth cranial nerve; SIH, spontaneous intracranial hypotension; th, thoracic. In SIH patients, the type of spinal CSF leak is classified according to Schievink et al.[18]
