# Supplementary figures and images for: Interrelation Between Cerebrospinal Fluid Pressure, Intracranial Morphology and Venous Hemodynamics Studied by 4D Flow MRI
Source: Clin Neuroradiol. 2024 Jan 26;34(2):391–401. doi: 10.1007/s00062-023-01381-0 (PMC11130051; doi:10.1007/s00062-023-01381-0)

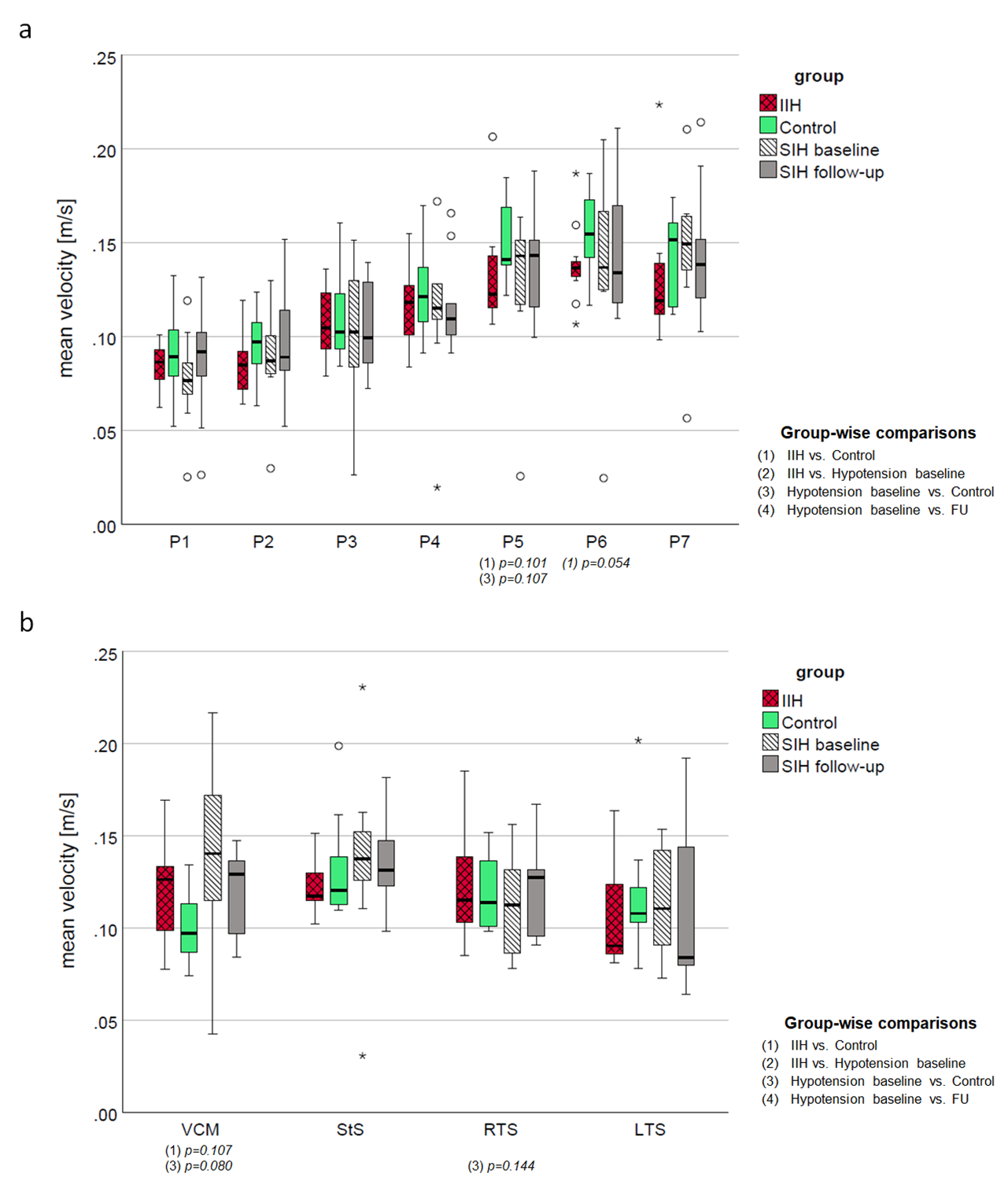

Supplement: Supplementary file 2 — Supplementary Fig. Boxplots depict venous flow velocity at cross-sections within the superior sagittal sinus (a; P1 is located rostral, P7 dorsal), and in the great cerebral vein (VCM), straight (StS), right (RTS) and left (LTS) transverse sinuses (b). Between-group comparisons did not show significant different velocities (simple t‑test). Longitudinal within-group changes in intracranial hypotension patients at baseline versus after treatment did not reach significance (dependent t‑test). Trends (italics) are indicated below the graph. FU follow-up, IIH idiopathic intracranial hypertension [file 62_2023_1381_MOESM2_ESM.tif]
